# Cyber Attack Detection for Self-Driving Vehicle Networks Using Deep Autoencoder Algorithms

**DOI:** 10.3390/s23084086

**Published:** 2023-04-18

**Authors:** Fawaz Waselallah Alsaade, Mosleh Hmoud Al-Adhaileh

**Affiliations:** 1 College of Computer Science and Information Technology, King Faisal University, P.O. Box 4000, Al-Ahsa 7057, Saudia Arabia; falsaade@kfu.edu.sa; 2 Deanship of E-Learning and Distance Education, King Faisal University Saudi Arabia, P.O. Box 4000, Al-Ahsa 7057, Saudi Arabia

**Keywords:** in-vehicle networks, controller area network, security, artificial intelligence, intrusion detection system

## Abstract

Connected and autonomous vehicles (CAVs) present exciting opportunities for the improvement of both the mobility of people and the efficiency of transportation systems. The small computers in autonomous vehicles (CAVs) are referred to as electronic control units (ECUs) and are often perceived as being a component of a broader cyber–physical system. Subsystems of ECUs are often networked together via a variety of in-vehicle networks (IVNs) so that data may be exchanged, and the vehicle can operate more efficiently. The purpose of this work is to explore the use of machine learning and deep learning methods in defence against cyber threats to autonomous cars. Our primary emphasis is on identifying erroneous information implanted in the data buses of various automobiles. In order to categorise this type of erroneous data, the gradient boosting method is used, providing a productive illustration of machine learning. To examine the performance of the proposed model, two real datasets, namely the Car-Hacking and UNSE-NB15 datasets, were used. Real automated vehicle network datasets were used in the verification process of the proposed security solution. These datasets included spoofing, flooding and replay attacks, as well as benign packets. The categorical data were transformed into numerical form via pre-processing. Machine learning and deep learning algorithms, namely k-nearest neighbour (KNN) and decision trees, long short-term memory (LSTM), and deep autoencoders, were employed to detect CAN attacks. According to the findings of the experiments, using the decision tree and KNN algorithms as machine learning approaches resulted in accuracy levels of 98.80% and 99%, respectively. On the other hand, the use of LSTM and deep autoencoder algorithms as deep learning approaches resulted in accuracy levels of 96% and 99.98%, respectively. The maximum accuracy was achieved when using the decision tree and deep autoencoder algorithms. Statistical analysis methods were used to analyse the results of the classification algorithms, and the determination coefficient measurement for the deep autoencoder was found to reach a value of R^2^ = 95%. The performance of all of the models that were built in this way surpassed that of those already in use, with almost perfect levels of accuracy being achieved. The system developed is able to overcome security issues in IVNs.

## 1. Introduction

Academics and businesspeople alike have taken an early interest in the emerging field of connected and autonomous vehicles (CAVs) [1]. In 2015, the government of the United Kingdom established the “Centre for Connected and Autonomous Vehicles” [2]. This organisation released a report in 2018 detailing R&D efforts in the field of CAVs. A study titled “Connected and Autonomous Vehicles: the future” [3,4] was released by the House of Lords in 2017. In 2017, several groups, including the British Standards Institute (BSI) in the UK, released reports detailing their plans to develop CAV standards [5].

**Figure 1:**
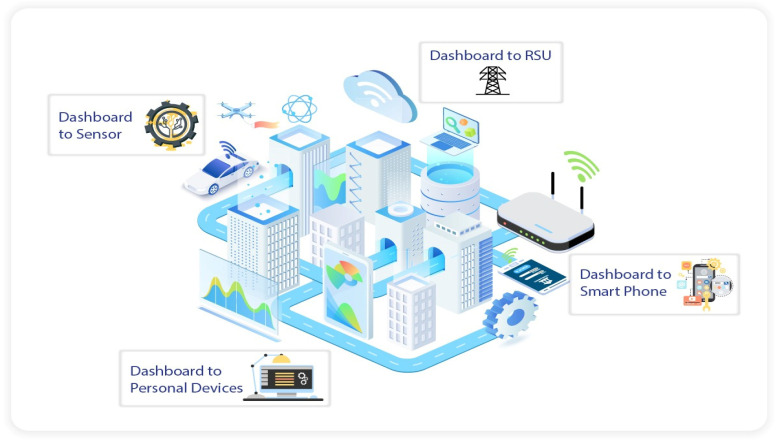
A smart city based on the IoT.

Autonomous systems such as self-driving cars will be commonplace in the future, and in preparation for their use, consumers and researchers will be able to practise using a variety of automation features that will be included in all future AV systems. Before being put into use, all of the features of an AV, including their functions and connections, safety alerts and privacy concerns, should be locked down. However, these capabilities can be compromised by cybersecurity vulnerabilities (CVs), resulting in either malfunctions or connections that just do not work. Cyberattacks, threats, and the mechanical failure of defective components, systems, and communication services that depend on energy efficiency all pose serious risks to the safety of AVs. Due to the fact that vulnerabilities in IoT devices also manifest in the context of data transmission, cybersecurity is rapidly becoming a more pressing issue in autonomous vehicles and other data communication systems. Figure 1 shows a smart city in which autonomous systems based on the IoT are used, revealing the pathways through which hackers can try to access car systems.

**Figure 2:**
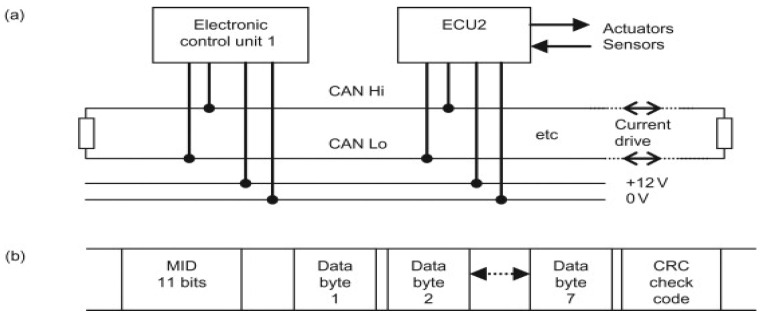
A CAN bus: (**a**) system connections and (**b**) frame format.

The high levels of in-vehicle capabilities seen in today’s intelligent automobiles are provided by electronic control units (ECUs). Furthermore, serial buses are used to link these components together. In today’s intelligent automobiles, the controller area network (CAN) protocol is in charge of ensuring that all of these components are able to effectively communicate with one another. Enhanced road safety, communication with the outside world, the plethora of new services designed to improve the customer experience, and so on, are just a few of the numerous benefits that contemporary in-vehicle technology enables intelligent cars with smart connectivity and computerisation to provide. On the other hand, hackers are able to more easily gain control of a vehicle’s systems due to the increased vulnerability introduced by these linked smart features. Access to the electronic components installed in today’s sophisticated automobiles might provide hackers with new avenues of attack [6,7]. Figure 2 presents a schematic representation of a CAN network.

Current in-vehicle network protocols contain several holes, such as insecure ID-based arbitration systems for conflict resolution and the lack of message authentication or encryption [8]. The security of today’s intelligent cars is a pressing concern, because there is a risk that attackers could exploit the flaws currently present to cause bodily harm or property damage on the road. Recent decades have seen the development of cutting-edge technology for autonomous cars, as well as other types of smart, intelligent car. There have been several recent successes in the automotive industry that can be directly attributed to the vast improvements in connectivity, as well as the proliferation of new communication channels and access points. On the other hand, new threats to data privacy and security have been made possible by these advancements [9]. As a result of these flaws in cybersecurity, human lives are in jeopardy. Given the increasing efficiency of the communication capabilities of the currently connected cars and the need for the real-time exchange of important safety-related information between vehicles and the surrounding infrastructure [10], it is clear that this is an area of rapid development. In light of this development, the landscape of automobile cybersecurity is changing rapidly and dynamically. Identifying potential security holes in network infrastructures as quickly as is feasible is crucial for effectively addressing cybersecurity concerns. There is unease that cybersecurity flaws [11,12] might compromise the safety of today’s intelligent automobiles when driving on the road. Those who work with unmanned aerial vehicles (UAVs), sometimes known as drones, face difficult challenges in terms of cybersecurity. To begin, because of the nature of CAV cybersecurity, it is difficult to anticipate all the different attacks that could take place. This presents a challenge. Everyone who creates software or uses it has a responsibility to be aware of the fact that attack patterns are also evolving, thus calling for a continuous response to the unknown. Those who attack CAVs only need to find a single opening through which to launch an attack. Furthermore, CAVs are assembled using a wide variety of components with specialised characteristics. If even a single one of these parts breaks, the integrity of the whole system will be compromised. Because of the linked nature of the many components that make up a CAV system, performing vulnerability assessments represents a challenging task. The many sensors found in CAVs produce enormous amounts of data, the administration of which is made more difficult by the fact that the data may be present in several different forms. If the data are in a format and include content that is consistent with the standards of the CAV, then the processing of the data will be much easier. In the end, CAVs are able to interact with one another owing to the use of a number of wireless communication technologies. These technologies include Bluetooth, dedicated short-range communications (DSRC) and WiFi, among others. Because of this, it is more difficult to guard against threats to cybersecurity in CAV networks than in wired ones.

There are several classification methodologies that use machine learning (ML) algorithms to better aid administrators in recognising network assaults, including the naive Bayes classifier, decision trees, logistic regression (LR), support vector classifiers (SVCs), and deep convolutional neural networks [13,14]. Algorithms like these can find application in a wide variety of categorisation approaches. In the future, OMIDS may need to be able to cope with a wider array of threats, including extortion, among other potential problems. The majority of ML methods that are currently available for the identification of cyberattacks on OMIDS were developed on the basis of cyber threat studies in which routing data were paired with prospective attack profiles gleaned through behavioural analysis techniques, such as packet collection, filtering and feature comparison. In these cyber threat studies, routing data were paired with prospective attack profiles. The routing information provided by CAN devices can be used to classify the many possible types of attack, assess the actual type of attack, detect which ECU has been hacked, and activate countermeasures. As a direct consequence of this, security managers have begun to use deep convolutional neural network techniques. The include long short-term memory (LSTM) networks [15], convolutional neural networks (CNNs) [16], and recurrent neural networks (RNNs) [17]. Deep convolutional neural networks (CNNs) provide a novel method for enhancing threat identification accuracy and decreasing false positives in network intrusion detection (FPR). In high-degree-of-freedom models, however, the high levels of variance (in other models) or large degrees of bias (in low-degree-of-freedom models) imply that it might not be possible to fit the data distribution well using a single base classifier [18].

Almost 1.3 million people are killed in automobile accidents, annually, as reported by the World Health Organization (WHO). Age is the single most significant determinant in mortality rate, with young people under the age of 29 being at highest risk of mortality. Driving under the influence of alcohol or drugs, driving while distracted, driving automobiles that present a high degree of risk, and driving in an unsafe environment are among the leading contributors to traffic crashes [5]. Excessive speeding is also a principal cause of traffic accidents. AVs support driving activities by sensing the surrounding environment, planning the quickest and safest routes, controlling speed, and performing navigation and parking without the input of a human driver. This helps to reduce the number of accidents caused by human error, which, in turn, helps to reduce the overall number of accidents. The possibilities arising from this have piqued the curiosity of a great number of scholars and businesses in a number of countries all around the globe. We can speculate as to the potential beneficial impacts that self-driving cars may have on both society and the economy, despite the fact that it is unlikely that they will find extensive utilisation in the near future. They have the ability to play a substantial role in decreasing the incidence of traffic accidents, rate of fuel consumption, and degree of congestion on roads. They have the capacity to do this in a number of different ways.

In addition, one of the aspects of CAVs that poses the greatest risk is their shortcomings in terms of cybersecurity, which can be a contributing factor in automobile collisions due to the fact that attackers are able to alter cars’ navigation systems. As a consequence of this, we came up with a plan to protect CAVs using an artificial intelligence (AI)-based security system that is able to recognise and repel any assaults directed at them. The stated fundamental purpose of the system is to ease the issues associated with information security in CAVs through the identification of possible attack messages and the implementation of CAV cybersecurity. The issue of robust construction is one that has to be addressed in order to combat cyber threats targeting IVN communication. In light of the fact that CAVs have emerged as a new technology in a number nations and have become ingrained in the fabric of everyday social life, it is vital to develop innovative methods for detecting infiltration using IVNs. The key aim of this study was the development of deep learning algorithms with the capacity to recognise intrusions into CAN buses located in automobiles. This method exhibits considerably improved accuracy of detection for various types of attack compared to the systems in place up until this point in time.

The primary objective of the presented system is to alleviate the difficulties associated with information security in CAVs via the detection of possible attack messages and the implementation of CAV cybersecurity. Data in the CAN bus protocol are not encrypted; therefore, hackers are able to perform replay attacks and inject malicious messages into networks (sometimes referred to as executing an intrusion-based attack) by employing a reverse-engineering technique in order to understand each CAN packet. This allows hackers to perform intrusion-based attacks. Therefore, in order to identify abnormal patterns of activity within vehicular networks, artificial intelligence technologies are necessary. In this article, we develop a prediction model that is able to identify unusual traffic patterns in the protocol of a vehicular network. This technology can be applied in the identification of traffic anomalies in automotive networks.

In the course of this research, we used a variety of machine learning methods, such the KNN, decision tree, and deep learning LSTM, and deep autoencoders approaches, in the construction of an effective intrusion detection system that is able to identify cyber threats to IVN communication. A deep autoencoding algorithm anomaly detection model is proposed with the aim of strengthening the CAN bus protocol used for in-vehicle network communication. The deep autoencoder is made up of two symmetrical deep-belief networks, each having four or five shallow layers. One of the networks serves as the encoding half of the net, while the other network is responsible for the decoding half; this combination makes it possible for the proposed deep autoencoder method to achieve efficient performance in terms of accuracy and the time cost of building the model. Finally, we demonstrate that a deep autoencoder with significant neural network parameters is appropriate for the detection of attacks with high accuracy on the basis of the CAN dataset, in contrast to our previous work.

## 2. Background Study

In this section, the principles underlying attacks on and vulnerabilities of IVNs will be described, as well as the cybersecurity methods utilised in their solutions. The majority of research efforts in this domain have so far been devoted to CVs and other mitigation measures for huge data transmission between roadside units (RSUs) and AVs. For instance, one of the more frequent kinds of attack, known as denial of service (DoS), may be avoided with the use of clever defensive techniques such as that which was recently reported. This method makes use of backpropagation neural networks to examine the percentage of overlapping scores. The proposed system safeguards external communications for autonomous and semi-autonomous vehicles, in contrast to current systems, and it does so without requiring the use of extra sensors, such as radar, LiDAR, computer vision or RSUs [19].

In the United States, some states have recently established laws that allow for the testing of CAVs on public roads [20]. The year 2009 marked the beginning of Google’s [20] work on the development of AVs. In 2016, the company established Waymo as a wholly owned subsidiary, and in 2018, it launched a pilot program in Phoenix in which a select group of locals were allowed to request driverless journeys (although a safety supervisor driver was still present in the vehicle). On the road, Tesla [21] has been developing autonomous driving solutions for CAVs and putting them into commercial use. A large amount of research has been published by academic institutions in the United States, one of which is the University of Michigan [22], which is situated in close proximity to an Mcity test area. Long-standing market leaders in Europe, including BMW, Audi and Mercedes-Benz, have all invested substantial amounts of money into the research and development of AVs [23].

In addition, the authors of [24] researched the flaws with respect to cybersecurity in AVs that make them vulnerable to sensor attacks. They proposed a novel rule-based intrusion detection system (IDS) for CAVs that was able to identify sensor attacks and localise their sources. The proposed inertial navigation system used a combination of a cumulative sum (CUSUM) discriminator and an extended Kalman filter (EKF) to estimate the vehicle’s location. Multiple sensors were installed, offering continuous updates on the state of the driverless car, making it safer from interventions from the outside world. In addition, a detector was employed to check for inconsistencies in the values being reported by the different sensors. Finally, the information gathered by the detectors was processed using a rule-based separation system to provide information on aberrant sensors. The developed model was presented in detail, together with the conclusions of the experiments, on the basis of which the model’s applicability for real-world data obtained from autonomous cars was demonstrated.

In addition, the authors of [25] investigated the classification of dangers in the context of autonomous automobiles, especially in relation to three essential security services: authentication, accountability and availability. In [26], the authors delved deeper into different defensive mechanisms against assaults on autonomous cars, as well as how such defensive mechanisms had developed during their investigation. In particular, the authors emphasised the significance of blockchains in resolving and mitigating privacy and security issues (for AVs). They wrapped up their investigation by discussing the actual problems and obstacles associated with using blockchain technology in AV security solutions.

The authors of [27] recommended the use of two different forms of deep learning to spot potential DoS attacks on electric vehicle charging stations (EVCSs). Python’s LSTM and deep neural network (DNN) algorithms were used by the authors to achieve the appropriate classification of distributed DoS attacks. It is thought that DoS attacks can be carried out by means of any network connection that is sufficiently weak. The DNN and LSTM algorithms were both trained, tested and confirmed by our team. According to the authors, both deep learning algorithms showed very high levels of accuracy.

The outcomes of the study that was conducted in [28] demonstrated that vulnerabilities in autonomous cars may put autonomous services in jeopardy. As a direct result of this, research has been conducted to classify the dangers posed by autonomous cars and to propose methods to combat these hazards. The authors proposed three unique types of attack, categorised on the basis of their target, which could be autonomous control systems, components of autonomous driving systems, or vehicle-to-everything communications. In [29], the authors provided a comprehensive review of cybersecurity as well as contemporary countermeasure strategies for the purpose of defending AVs and the services that they supply. Furthermore, that study also covered the requirements for CAVs, as well as the unresolved challenges that still need to be addressed. According to the findings of yet another study [30], the security of autonomous driving can be broken down into four distinct categories: sensors, operating systems, control systems and vehicle-to-everything connections. ECUs, sensors, intra-vehicular connections, and inter-vehicular linkages are all potential points of attack, and in [31], the methods of defence against such attacks were modelled and discussed. In [32], a high-level description of security problems related to data transmission was provided across a range of apps, including antivirus programs, in addition to solutions for mitigating these flaws. The primary focuses in [33] were cybersecurity for robots and the development of security solutions making use of multi-factor cryptographic approaches in order to guarantee the safety of autonomous systems. Multi-access edge computing (MEC), which was studied in [34], can be used to uncover vulnerabilities in the security of 5G-based use cases (AVs may benefit from MEC, since it can improve the performance of automated driving, augmented reality and machine-type communications). Another study [35] provided detailed information on the use of autonomous systems to assist in the development of upcoming autonomous mobility services. CAVs make use of a wide array of IoT sensors in order to keep an eye out for any dangers in their immediate environments. In [36], the authors presented a security policy road map as a means of attaining long-term AV adoption. The policy packages that are meant to actualise superblock vision consist of six overarching themes, and these topics describe the measures that need to be taken in order to achieve the changes envisioned in transportation legislation by the year 2050. The goal of [37] was to determine how to achieve the highest possible levels of safety and security while integrating AVs with intelligent transportation systems (ITSs). Because the transportation systems of the future will be very complicated and highly dependent on a wide range of distinct technological fields, we anticipate that they will be adaptable, dynamic and open to changes and improvements with respect to the level of safety they provide. By the year 2030, it is anticipated that the implementation of four distinct types of AI countermeasures, namely those that are AI-supplemented, AI-generated, AI-mediated and AI-facilitated, will assist in the resolution of selected security concerns in autonomous transportation services, namely CVs, and countermeasures for data transmission. As a direct consequence of this, it will be necessary to satisfy more stringent regulations [38,39,40,41,42,43] in the planning and development of autonomous transportation services.

There is a paucity of published material dedicated to the use of and research into CTI on IoV; maybe this is because IoV technologies are still relatively novel and immature, and are seldom utilised in actual practice at this stage. According to Kukkala et al. [44], in order to effectively combat cyberattacks in CAVs and IoV systems, it is important to employ threat intelligence to achieve enhanced cybersecurity testing and to disseminate this knowledge across diverse industries. In a similar vein, He et al. [45] highlighted the benefits of using AI methodologies, such as ML, to analyse CTI in IoV systems. These benefits include the capacity to manage vast volumes of data and to automatically carry out tasks. Honeypots were recommended by Panda et al. [46] as a method for gathering and analysing CTI in IoV situations. These are designed to deceive attackers. Basnet et al. [47] used deep learning algorithms as an example to illustrate how CTI may be used to detect cyberattacks in CAVs. CTI stands for cyber threat intelligence. Ali et al. [48] proposed the use of several ML algorithms to provide secure communication between vehicles. In addition to safety, the preservation of the environment and maintaining stability are also quite important. Liu et al. [49] proposed cloud-edge computing for performing IoV service deployment and execution with privacy protection. Mohseni et al. [50] studied realistic ML safety solutions that could potentially be used to enhance engineering safety for ML-based software in autonomous automobiles. This is essential work, since engineering safety for ML-based software in autonomous cars is critical. A paradigm in which CTI was modelled and domain-specific hazard categories were defined was suggested by Kumar et al. [51] and applied in the setting of maritime transportation systems, which is a relevant field of study.

As a result, the gap detected in previously conducted research may be summed up as follows: To begin with, there is no single method that can be used to systematically evaluate the possible weaknesses of CAVs to multiple attacks. While most studies have focused on the theoretical aspects of CAV cybersecurity, or the use of machine learning and deep learning, the detection rate has remained low due to the complexity of CAN networks. Therefore, we aimed to enhance these algorithms to increase the detection rate.

In this article, we will discuss some of the most recent findings regarding intrusion detection in IoV. By observing typical traffic patterns in an intra-vehicle network, V2V communications, and V2I networks, in [51,52], the authors introduced a DL-based IDS for ITS. The LSTM autoencoder is the foundation of the proposed IDS, and is able to detect abnormal events in IoV at the primary gateway. The model was tested for inter-vehicle communications using car-hacking. The primary motivation for developing this approach was to improve system performance while simultaneously minimising computing requirements. We developed a system that is able to improve the detection rate to >99% in terms of the accuracy metric.

The purpose of this article is to construct an AI-based CAV framework in order to conduct an analysis of the potential cybersecurity risks posed to CAVs using the UK CAV cybersecurity framework as a guide to assist in the creation of a methodological approach for safeguarding CAV systems and the data that are transmitted among them.

## 3. Materials and Methods

**Figure 3:**
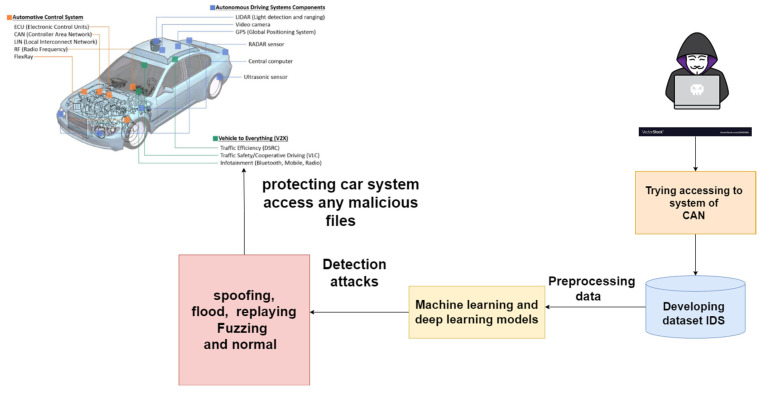
Framework of a CAN-based IDS.

Figure 3 displays the framework of a CAN-based IDS using ML and deep learning algorithms. Python was used to implement the proposed model on a PC with an Intel Core i7 2.30 GHz processor and 8 gigabytes of random-access memory.

### 3.1. Dataset

#### 3.1.1. Car-Hacking Dataset

Our experimental datasets were generated using data from standard datasets. The experiment employed a pair of specialised Raspberry Pi devices: one to record data from the network and one to serve as the attack node and inject fake data. The system had a link to the car’s internal systems. The car was connected to the network using its OBD-II connection (which is often found under the dashboard). The bespoke nodes were able to communicate with genuine ECU nodes across the CAN bus via the OBD-II port. Under typical conditions, the CAN bus is able to recognise 26 unique CAN IDs. The proportions of injected messages to total messages in each experimental dataset is detailed in Table 1.

The datasets contain four types of attack, namely, distributed denial of service attacks, fuzzy attacks, drive gear spoofing, and RPM meter spoofing. Each dataset was generated by recording CAN traffic while injecting artificial messages in a lab setting. The vehicle was left in a stationary position with the engine running while readings were taken. A total of 300 unauthorised messages were included in each dataset. The average duration of each incursion was 3–5 s, while the total amount of CAN traffic in each dataset was between 30 and 40 min. Every 0.3 ms, we launched a denial-of-service attack on the CAN bus by injecting messages with a CAN ID consisting of 29 zeroes. The goal of a denial-of-service attack is to reduce the usability of a network. Since the arbitration phase always favours the ECU attempting to send a message that has the most dominant CAN ID, the other ECUs are unable to send their messages. While the CAN ID and data values of messages are fixed in a DoS attack, they are completely unpredictable in a fuzzy assault. Every 0.5 ms, completely random messages were injected. The goal of the fuzzy attack is to cause the car or truck to act up. The driving gear and RPM gauge datasets were spoofed by injecting signals with a certain CAN ID every 1 millisecond. These alerts detailed the gear ratio and RPM readings, respectively. Spoofing attacks trick the vehicle’s original ECU into thinking that the RPM readout and drive gear have been altered. It was decided that the best CAN packet generator would be the open car testbed and network experiments (OCTANE). The injection attacks on the CAN traffic are presented in Table 2. The dataset contained 806,390 instances with four attacks and normal. The variables of the dataset are presented in Table 3.

#### 3.1.2. UNSW-NB15 Dataset

In a Cyber Range Lab at the University of New South Wales (UNSW), Canberra, the IXIA PerfectStorm program was used to simulate both everyday real-world activities and advanced persistent threats. The tcpdump program was used to record one hundred gigabytes of unprocessed data flow, which was later evaluated (e.g., Pcap files). Fuzzers, analysers, backdoors, denial-of-service attacks, exploits, generic reconnaissance, shellcode, and worms were just some of the types of injection attack included in this dataset. Figure 3 demonstrates how 49 characteristics were generated using the Argus and Bro-IDS programs with a total of 12 algorithms. Table 1 provides a summary of the attacks dataset.

### 3.2. Pre-Processing Method

The CAN traffic data are very complex; therefore, the pre-processing step is very important for improving the classification algorithms. The dataset contains features such as DLCs, which have hexadecimal characteristics and three label classes, namely flooding, spoofing and replay; these convert categorical variables into numeric variables to help the proposed system to detect attacks. One-hot encoding is one of the most frequently used methods for obtaining numerical values by converting categorical characteristics.

After the categorical variables were converted, maximum–minimum normalisation methods were utilised in order to prevent any potential overlap in the training process brought on by the manipulation of large datasets. During the normalisation process, we scaled the dataset using a scaling range of 0 to 1 in order to keep everything within the same range.

(1) $$Normalisation=\frac{y-y_{min}}{y_{max-y_{min}}}\left(x_{{max}_{x}}-x_{{min}_{x}}\right)+x_{{min}_{x}}$$

where $y_{min}$ and $y_{max}$ are the minimum and maximum data values.

### 3.3. Machine Learning Algorithms

#### 3.3.1. k-Nearest Neighbour (KNN)

The k-nearest neighbour (KNN) method is a basic, easy-to-implement supervised ML technique that may be used to tackle both classification and regression problems. Its name comes from the fact that it finds the neighbours that are closest to each data point.

The KNN algorithm is an ML method that examines the immediate neighbours of a data point in order to discover connections between the points. The algorithm finds groups or clusters of similar data by first computing the closest distance between each pair of data points and then using that knowledge to find groups or clusters of similar data. This process is repeated until the algorithm has found all such groups or clusters. KNN can be used in a variety of applications, including for solving classification problems (where it is widely used as an alternative to traditional linear regression techniques) and hyperparameter optimisation problems. KNN was developed in the 1980s, and has been improved upon significantly since then. KNN is frequently more accurate than other algorithms when working with enormous datasets; the fact that it is so simple makes it applicable in the context of a wide variety of issues. In this investigation, we determined the distances between different classes of ICS network data by employing a function called Euclidean distance (E_i_). To express the Euclidean distance function mathematically, the following formula was used:

(2) $$E_{i}=\sqrt{\left(c_{1}-c_{2}\right)+(d_{1}-d_{2}{)}^{2}}$$

where c_1_, c_2_, d_1_ and d_2_ are the input data variables.

#### 3.3.2. Decision Trees

The decision tree (DT) component of ML approaches is a commonly used strategy for addressing classification and regression problems. In DT models, a root node is located at the very top, and branches that are dependent on the data’s essential characteristics are located at the very bottom. An output branch represents the output of a feature, whereas an output child node represents the output of a category in a tree structure. One method of learning the classification model is by employing a classification DT, which is an illustration of supervised learning. This method relies on sample training. In the end, the process of classification concludes when the incoming data, which are assessed by each node, have all been considered. There are three different types of decision tree, which are classified according to the parameters that are used to define the attributes of the branch nodes: ID3, C4.5 and CART. ID3 makes use of a greedy method, and which branches to take is determined on the basis of information *entropy* [48].

(3) $$Entropy=\left(S\right)=\sum_{i=1}^{C}p_{i}{log}_{2}p_{i}$$

(4) $$entropy\left(S|B\right)=\sum_{j=1}^{j}\frac{\left|s_{i}\right|}{\left|S_{i}\right|}entropy(S_{i})$$

(5) $$\mathrm{Gain}\;\left(S|B\right)=entropy\left(S\right)-entropy\left(S|B\right)$$

where S is the training dataset and the class of the CAN dataset is denoted by C, which is either attacks or normal. $P_{i}$ is the probability of simple data indicating class $S_{i}$, which is the simples of subsets of a class in feature *B*.

### 3.4. Deep Learning Models

#### 3.4.1. Long Short-Term Memory (LSTM)

**Figure 4:**
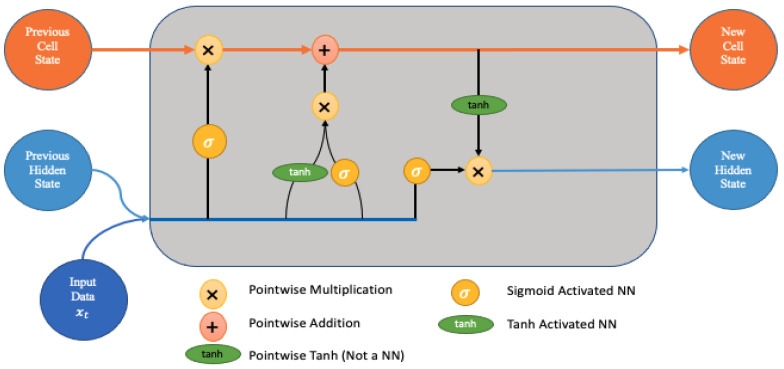
LSTM structure.

Long short-term memory (LSTM) networks are a form of RNN that can learn order dependency. These networks are useful for solving difficulties involving sequence prediction. In RNNs, the input for the current step is taken from the output of the step that came before it [52,53,54]. LSTM was developed by Hochreiter and Schmidhuber. It addresses the problem of the long-term reliance of RNN, whereby RNNs are unable to predict words that are stored in long-term memory but are able to make more accurate predictions on the basis of data that are available now. With increasing gap length, the RNN’s performance degrades. Long short-term memory (LSTM) is designed to save data for a very long period. The building blocks of an LSTM consist of four neural networks and several memory nodes connected in a chain configuration. A typical LSTM unit consists of a cell, an input gate, an output gate, and a forget gate. The information that enters and exits the cell is regulated by three gates, and the cell is able to retain data for an indefinite amount of time. Figure 4 shows the structure of an LSTM model.

The most fundamental building block of an LSTM network is referred to as a component, the constituent parts of which are the cell, the input gate, the output gate, and the forget gate. It is the responsibility of the cell to remember values at any given moment, and the three gates of the cell are responsible for controlling the flow of information into and out of the cell. What follows is a brief summary of the three different entryways: (1) One of the gates is called the “forget” gate, and is responsible for either erasing or retaining information, depending on the current input and the state that was concealed before. In order to arrive at a conclusion, everything is first taken into consideration. The sigmoid function is used to evaluate these integers, and the results are always in the range of 0 to 1. If the value is closer to zero, the item in question is to be forgotten, but if it is closer to one, the item in question is to be kept. (2) Input gate: The input gate determines which individual pieces of information will be retained as component parts of the cell state. Both the sigmoid function and the tanh function are used in the process of bringing the data up to date. It is the responsibility of the sigmoid and tanh functions to determine which aspects of the data need modification. Eventually, the cell state is revised on the basis of the values resulting from these procedures. (3) Output gate: This gate determines the final output by using the sigmoid function to select valuable information from the current cell state as the output, while the tanh function is used to acquire the final output. The final output is determined by this gate. RLSTM gates can be represented mathematically, as reported in [47,48], as shown below:

(6) $$f_{t}=\sigma \left(W_{f}.X_{t}+W_{f}{.h}_{t-1}+b_{f}\right)$$

(7) $$i_{t}=\sigma (W_{i}{.X}_{t}+W_{i}{.h}_{t-1}+b_{i})$$

(8) $$S_{t}=tanh(W_{c}{.X}_{t}+W_{c}{.h}_{t-1}+b_{c})$$

(9) $$C_{t}=(i_{t}*S_{t}+f_{t}*S_{t-1}$$

(10) $$o_{t}=\sigma (W_{o}+X_{t}+W_{o}{.h}_{t-1}+V_{o}.C_{t}+b_{o})$$

(11) $$h_{t}=o_{t}+tanh(C_{t})$$

where f is a forget gate, sigmoid is a function, $W_{f}$ is a weight between the forget gate and the input gate, $h_{t}$ is the previous hidden state, $i_{t}$ is the input at the current timestamp, it is the input gate at time t, Wi is the weight of the respective gate, tanh is the tangent function, $W_{c}$ is a weight between the cell state and the network output, where the cell state is represented by $C_{t}$, the output gate weight by $W_{o}$, the input and output biases by $b_{f}$, $b_{i}$ and $b_{o}$, respectively, and the LSTM output by $h_{t}$.

#### 3.4.2. Deep Autoencoder Algorithms

**Figure 5:**
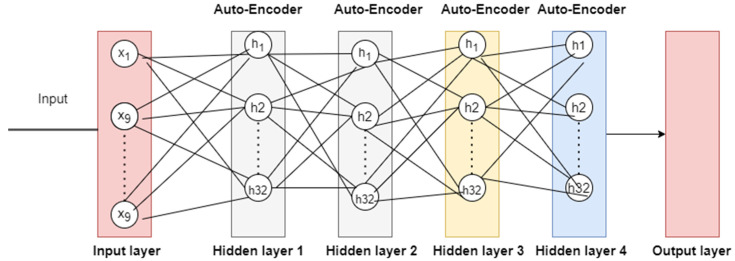
Autoencoder algorithm structure.

Encoders and decoders are important components of the autoencoder approach. An encoder component takes the incoming data and converts it into the most simplified form possible. The lowest data representation from the encoder is used by the decoder to faithfully recreate the input data. The processing of data includes encoding and decoding steps. Autoencoders encode all data coming from the input layer, transmit it through many layers of hidden processing, and then decode it before delivering it to the production layer (output layer) of the network. The recognition accuracy of autoencoder-based deep learning models for IDSs may be very sensitive to the design and hyperparameter settings of the autoencoder model. This is due to the versatility of autoencoders in terms of their ability to uncover many forms of assault. Therefore, it is crucial to identify the optimal parameters of autoencoders for enhancing detection accuracy. Previous mainstream studies have detailed methods for selecting the best model for each unique situation by testing several configurations on specific datasets. Human-method testing for intrusion detection is time consuming, and must be repeated whenever the data are updated. When applied to intrusion detection systems (IDSs), the deep autoencoder (DAE) model is a two-step process that may address IoT network security concerns. These methods also include training and testing. In order to train a classifier, the system utilises the dataset acquired by the selected DAE. To determine which class each sample in the test dataset falls into, IDSs use autoencoder models. In this way, the IDS may evaluate how well the system functions when it is put to use in a networked environment. Figure 5 shows diagram of the suggested DAE architecture for intrusion detection. There are three separate layers in this architecture: the input layer, the hidden layer, and the output layer.

The complexity of CAN messages in the dataset is considerable; this causes the dataset to be unbalanced, thus increasing the time cost of detecting the attacks using deep leaning and machine algorithms.

### 3.5. Performance Measurements

It was recommended that the high performance of the CAN security system be evaluated on the basis of a variety of metrics, including, precision, recall, F1 score, mean square error (MSE), determination coefficient (R^2^), and root mean square error (RMSE). The following are examples of such measurements:

(12) $$MSE=\frac{1}{n}\sum_{i=1}^{n}{\left(y_{i,target}-y_{i,pred}\right)}^{2}$$

(13) $$RMSE=\sqrt{\sum_{i=1}^{n}\frac{{\left(y_{i,target}-y_{i,pred}\right)}^{2}}{n}}$$

(14) $$R^{2}bn1-\frac{\sum_{i=1}^{n}({y_{i,target}-y_{i,pred})}^{2}}{\sum_{i=1}^{n}({y_{i,target}-y_{avg,target})}^{2}}$$

(15) $$Accuracy=\frac{TP+TN}{TP+FP+FN+TN}\times 100\%$$

(16) $$Recall=\frac{TP}{TP+FN}\times 100\%$$

(17) $$Precision=\frac{TP}{TP+FP}\times 100\%$$

(18) $$Fscore=\frac{2*preision*Sensitivity}{preision+Sensitivity}\times 100\%$$

where FP, FN, TP, TN are false positive, false negative, true positive and true negative, respectively, $y_{i,target}$ represents the target values, while $y_{i,pred}$ corresponds to the predicted values.

## 4. Experiments

Python was used to implement the proposed models in a PC with an Intel Core i7 2.30 GHz processor and 8 gigabytes of random-access memory. The experimental setup consisted of three steps: (1) the pre-processing phase; (2) the training process for the ML and deep learning models; and (3) the validation and evaluation step. Subsequently, each of the pre-processed datasets was split into separate sets of training data and test data. The pre-processed data were entered separately as components into the ML and deep learning models, on the basis of which an accurate feature representation of the data was extracted in accordance with their respective specialisations. The proposed deep-learning-based system consists of input data comprising three features and five classes, a hidden layer consisting of 520 neurons that has been trained using a deep learning model, and a hidden layer with 256 neurons. We used a grid search strategy to determine the optimal learning rate in order to obtain the best possible outcomes. The learning rates used were 0.01 for the batch size of 0.001 and 20 for the larger batch size. The training consisted of a number of epochs with a minimum value of 1 and a maximum value of 100. In order to bring the weightings in the LSTM network up to date, an adaptive moment estimation optimiser, or Adam, was employed. The learned traits were categorised using the SoftMax activation function, which was also used to determine whether intrusive behaviours were typical or unusual. Cross-entropy was used as the loss function in order to achieve higher levels of efficiency. These parameters were selected because they produced the best results on the basis of the preliminary experiments performed. The pseudocode of the LSTM model is presented in Algorithm 1 for reference.

### 4.1. Results

**Figure 6:**
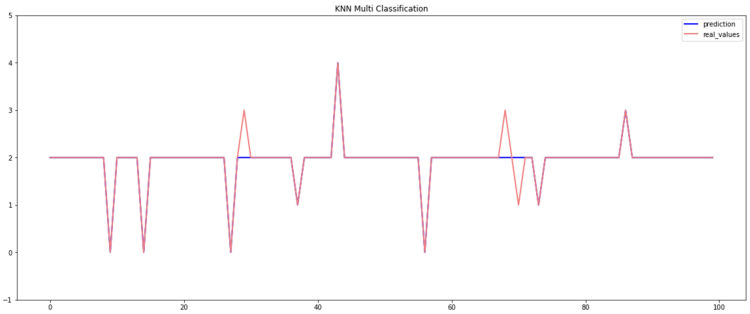
Performance of the KNN model (Car-Hacking dataset).

**Figure 7:**
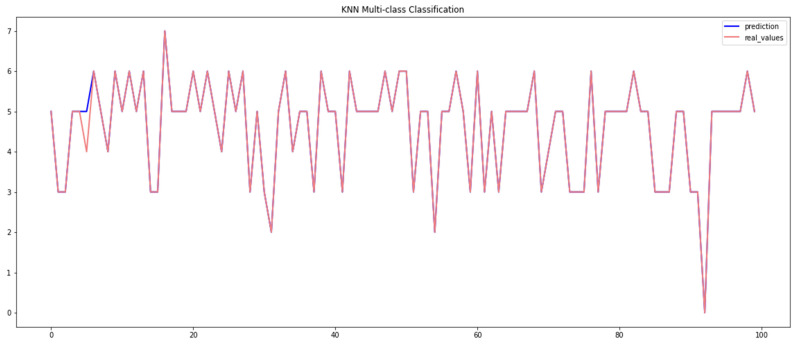
Performance of the KNN model (UNSW dataset).

Table 4 displays the results obtained by the KNN algorithm when using the Car-Hacking and UNSW datasets, which demonstrate that the KNN method attained an overall accuracy of 98.80% when using the Car-Hacking dataset, while it achieved an accuracy of 97.37% when using the UNSW dataset. However, KNN performed poorly when identifying spoofing (gear) attacks in the Car-Hacking dataset, and an even lower accuracy when identifying backdoor attacks. The effectiveness of the KNN approach for detecting breaches can be seen in Figure 6 and Figure 7 for both datasets.

**Figure 8:**
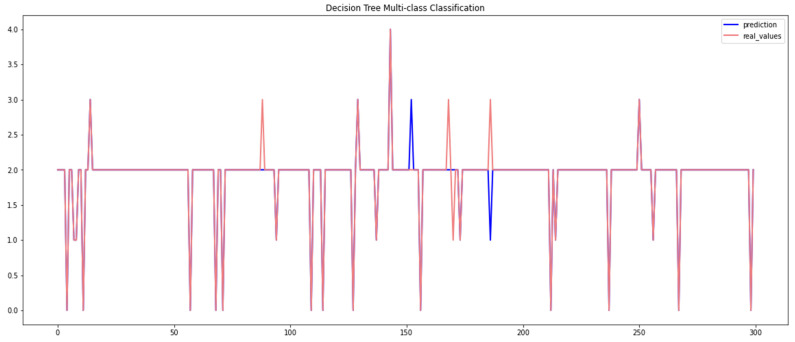
Performance of the decision tree algorithm (Car-Hacking dataset).

**Figure 9:**
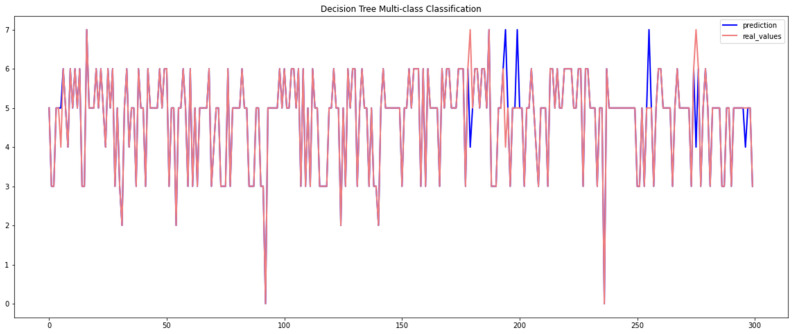
Performance of the decision tree algorithm (UNSW dataset).

Table 5 presents the empirical results obtained by the decision tree algorithm when using the Car-Hacking and UNSW datasets. It can be observed that this algorithm scored a high level of overall accuracy (99%) when using the Car-Hacking dataset, despite only having low accuracy when detecting spoofing (gear) attacks. The performance of the decision tree model when using the UNSW dataset was accuracy = 97.19%. The performance of the decision tree algorithm is presented in Figure 8 and Figure 9.

#### Results of the LSTM and Deep Autoencoder

The proposed deep learning methods, including the LSTM and deep autoencoder models, were used to identify attack signals emanating from the vehicle network. In order to test the system, a genuine network was applied to it, consisting of flooding attacks, fuzzy attacks, spoofing attacks, replay attacks, and regular traffic. It was decided to utilise 80% of the dataset at random for training and the remaining 20% for testing. During the training phase, the system database comprised 486,640 packets, and the same number of messages were used in the database during the testing phase. The results of the deep learning models’ attempts to detect attacks are presented in Table 6. The LSTM model successfully achieved good values in the areas of precision (96.18%), recall (96.17%) and F1-score (96.82%) when using the Car-Hacking dataset, while when using the UNSW dataset achieved an accuracy of 98.69. The autoencoder model achieved an overall performance of 99.98% with respect to identifying attack messages originating from CAN busses when using the Car-Hacking dataset. Meanwhile, it achieved an accuracy of 98.09% when using UNSW dataset.

**Figure 10:**
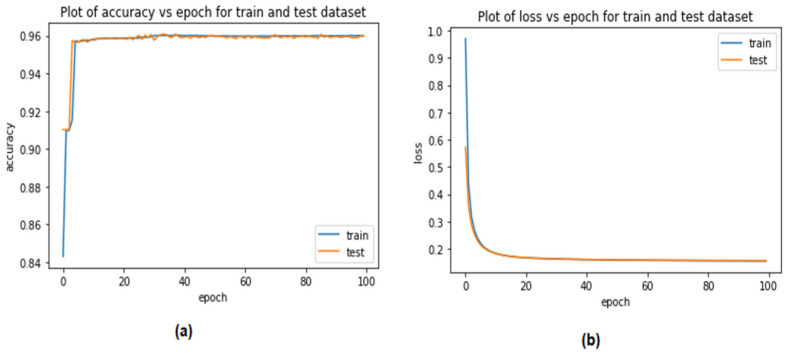
Performance of the LSTM model when detecting CAN attacks (Car-Hacking dataset) (**a**) validation accuracy (**b**) validation loss.

**Figure 11:**
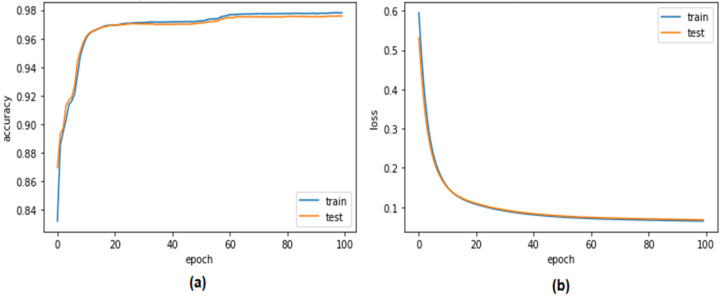
Performance of the LSTM model when detecting CAN attacks (UNSW dataset) (**a**) validation accuracy (**b**)validation loss.

Figure 10 and Figure 11 illustrate the performance of the proposed LSTM system in terms of its level of accuracy when using the Car-Hacking and UNSE-NB15 datasets. The percentages of errors that were found and fixed are shown on the y-axes. The effectiveness of the validation mechanism is referred to as the training accuracy. In an effort to improve accuracy over the course of 100 epochs, the system halted the optimisation procedure. The LSTM model’s accuracy jumped from 84% to 96.03% when using the Car-Hacking dataset. Meanwhile, the accuracy LSTM model during validation when using the UNSW-NB15 dataset increased from 85% to 97.82%. On the basis of extensive testing, it was determined that the categorical cross-entropy function was the optimal method for gauging the training loss of the LSTM using the Car-Hacking dataset. The LSTM loss is shown in Figure 10b. After 100 epochs, it was also discovered that the validation loss decreased from 1.0 to 0.2, while the training loss decreased from 0.6 to 0.2. Both of these changes occurred simultaneously. The validation loss of the LSTM model when using UNSW-NB15 was 0.2.

**Figure 12:**
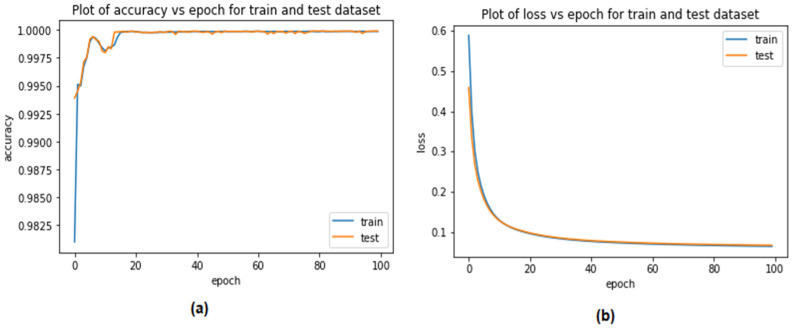
Performance of the autoencoder when detecting CAN attacks (Car-Hacking dataset) (**a**) validation accuracy (**b**) validation loss.

**Figure 13:**
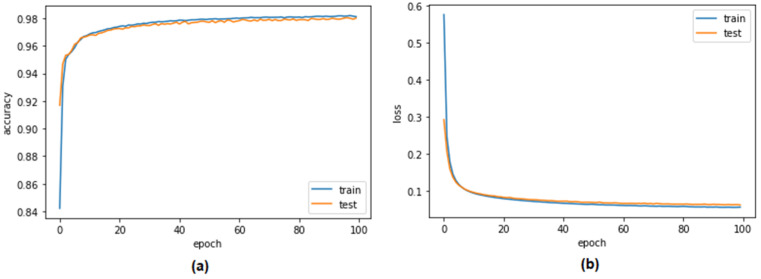
Performance of the autoencoder when detecting CAN attacks (UNSW dataset) (**a**) validation accuracy (**b**) validation loss.

As shown in Figure 12 and Figure 13, one of the evaluation criteria was the validation performance of the autoencoder model for distinguishing CAN attacks from typical packets on a CAN bus when using the Car-Hacking and UNSE-NB15 datasets. This constituted one of the evaluation criteria. It was hoped that the model would be able to discriminate between the two distinct types of packets. The accuracy of the system’s validation increased from 98% to 99.98% over a period of 100 epochs of testing when using the Car-Hacking dataset. Meanwhile, accuracy of the autoencoder model when using the UNSE-NB15 dataset was 98.09%. Because the overfitting of the system was extremely minor, the validation loss was quite small. Utilising cross-entropy measures helps to bring the validation loss down to 0.1, representing a significant improvement to the autoencoder model when using both datasets.

Dataset statistics, such as mean square and root mean square errors and the squared metrics for the specific properties of the datasets, are shown in Table 6. The statistical analysis revealed a substantial gap between the descriptions of the qualities and the descriptions of their labels. We discovered that the conventional methods used to identify attack messages in a CAN system are not suitable for use in this context.

Mean absolute error, mean square error, root mean square error, and correlation coefficient were also used to determine the degree of discrepancy between the predicted values and the actual values (R^2^). A statistical analysis of ML and deep learning is presented in Table 7, below. The decision tree method exhibited the strongest correlations between the target and predicted values, with an R2 value of 94.70% and a prediction output of 0.01190.

**Figure 14:**
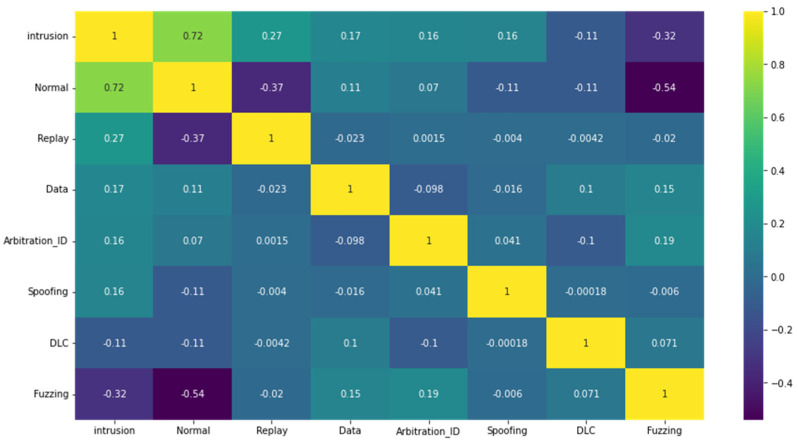
Correlation between features and attacks (Car-Hacking dataset).

The relationships that exist among the dataset’s characteristics can be seen in Figure 14. Because of the numerous distinguishing aspects of the network, there is a disparity between the features. Using Pearson’s correlation coefficient, the findings of our investigation led us to conclude that there was a significant connection between the qualities of the input and class membership. Certain characteristics showed substantial links. We chose characteristics that had a significant number of links with classes.

## 5. Discussion

Autonomous vehicles (AVs) have the potential to bring about a wide range of advantageous changes, such as an improvement in overall safety and reductions in energy consumption, pollution and traffic congestion. If security and privacy concerns are not addressed; however, the advantages that are anticipated to result from the use of these engines may not be realised. Hackers will be presented with additional opportunities to carry out destructive attacks thanks to AVs, which could pose a significant risk to the future of mobility and data security.

Controller area networks (CANs) have not yet integrated sufficient security methods, such as message authentication and encryption, despite their being the most extensively used in-vehicle communication protocol. Consequently, the CAN bus is susceptible to a wide variety of cyberattacks. It would seem that the use of the Internet of Things (IoT) in the sphere of transportation has enormous untapped potential. Cars equipped with intelligent vehicle systems are able to share information with one another in a smooth manner, helping to improve both traffic management and road safety. The dynamic architecture of this network connects a huge number of cars, making it susceptible to a variety of attacks, including those related to authentication, data integrity and confidentiality. These dangers put the well-being of passengers, as well as vehicles and the system as a whole, in jeopardy. In order to protect connected and autonomous cars against potential vulnerabilities, researchers have explored a variety of strategies.

A wide variety of intrusion detection systems (IDSs) have been created specifically to identify and stop these kinds of attacks. AI-based IDSs, on the other hand, are an ideal defence mechanism against cyberattacks on automotive systems due to the tremendous generalisation capabilities of AI.

**Figure 15:**
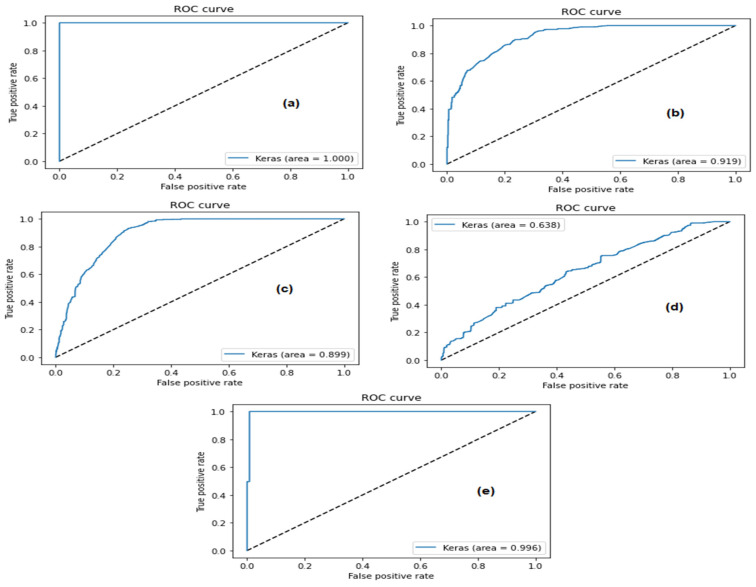
ROC of the LSTM model for (**a**) flooding attacks, (**b**) fuzzy attacks, (**c**) regular traffic, (**d**) spoofing attacks (gear) and (**e**) spoofing attacks (RPM) (Car-Hacking dataset).

The method proposed in this research uses ML and deep learning models to detect attacks on the CAN and to protect the network. The system was tested using a standard dataset. The deep autoencoder scored a high accuracy level of 99.98%. Figure 15 shows the receiver operating characteristic (ROC) curve of the LSTM model for the detection of CAN attacks.

Table 8 presents a comparison of the performance of the proposed machine and deep learning models for intrusion detection in CAN networks between the Car-Hacking and UNSW-NB15 datasets. It can be observed that that the proposed system achieved high performance when using the Car-Hacking dataset.

**Figure 16:**
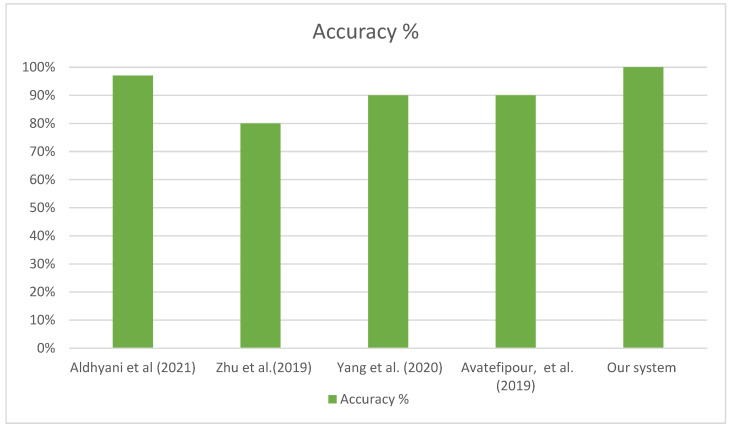
Comparison between the accuracy of the proposed system and existing CAN security systems [52,55,56,57].

In Table 9, a comparison of the accuracy of currently available deep learning algorithms determined by other researchers, and which are considered to be state-of-the-art models, with our obtained findings is the presented. The comparisons demonstrate that our suggested model is superior in every respect, particularly with regard to the precision of the data generation process. The accuracy of the suggested framework, which achieved a score of 99.98%, beat all existing systems in terms of its ability to identify threats to vehicle networks. Figure 16 shows how the suggested solution fares in terms of accuracy compared to other setups for CAN security.

## 6. Conclusions

The rapidly advancing state of computing has had a considerable influence on the research and development of AVs in a variety of domains. Because of the crucial roles they play in enhancing the quality of life in established cities, autonomous cars are increasingly being recognised as an essential component of the infrastructure required for the development of smart cities. Despite this, driverless cars are vulnerable to a wide variety of cyberattack vectors, which puts the lives of people in jeopardy. Therefore, the purpose of this work was to design, create and evaluate an intelligent anomaly detection system for autonomous cars. In this study, we propose an IDS that is able to detect anomalies in the CAN bus in intra-vehicular networks using ML models. In this study, an in-depth discussion of the vulnerabilities of the CAN bus was presented, along with the reasons for which AIDS is necessary in this particular field and the different categories of attack that CAN busses may be subjected to, as well as the effects such attacks may have on cars and drivers.

Therefore, the purpose of this research was to investigate potential security flaws arising from the presence of adware and malware inside autonomous car systems. In order to identify potentially harmful activities taking place in the network in the module, the network traffic was studied. In addition, an intrusion detection module based on ML and deep learning was presented for the detection of malware. This module uses techniques such as the k-nearest neighbour (KNN) and decision tree, long short-term memory (LSTM) and deep autoencoder methods. In conclusion, an ML system was presented that, with a high degree of precision and in a very short amount of time, is able to identify malware in automobiles. Metrics such as mean square error (MSE), root mean square error (RMSE) and correlation coefficient (R2) were assessed during the validation phase. This investigation led to the identification of the errors that persist between the expected output and the target values. The prediction errors generated when using the KNN and decision tree, LSTM and deep autoencoder algorithms were greatly reduced when used for binary classification and multi-class classification.

We evaluated the accuracy of the suggested system by comparing it to the accuracy of currently available systems. In addition, we discovered that the deep autoencoder method achieved a high level of accuracy (99.98%) when using the Car-Hacking dataset and an accuracy of 98.09 when using the UNSW-NB15 dataset, when tested against a variety of current systems, thus demonstrating that it is appropriate for real-time malware identification in scenarios involving AVs. The empirical findings demonstrate that the suggested algorithms are capable of recognising attack messages. The proposed systems were shown to be able to effectively detect anomalous packets in order to safeguard the CAN bus. They may also be extended to the design of various security system housed within the complex infrastructures of networks that characterise AVs in order to provide safe data processing. Our system will continue to evolve with the help of cutting-edge AI in the near future.

## Figures and Tables

**Table 1:** Total numbers of injected messages for each attack.

Attacks	#Message	Normal Messages	Injected Messages
#Flooding_attack	3,665,771	3,078,250	587,521
#Fuzzing_attack	4,443,142	3,845,890	597,252
#Normal	4,621,702	3,966,805	654,897
#Spoofing (gear)_attack	3,838,860	3,347,013	491,847

**Table 2:** CAN bus attacks.

Attack	Description
Flooding Attack on the CAN	Delivery of a large number of messages simultaneously from the CAN to the various ECU nodes. Attacks were injected at a rate of one every 0.3 milliseconds.
Spoofing (gear) Attack on the CAN	The network assault known as a spoofing attack occurs when cybercriminals recognise and detect a data transmission, after which they either delay or replay the transmission. The cyberattacker either causes the data transfer to be delayed or causes it to be repeated.
Spoofing Attack (RPM/gear) on the CAN	Spoofing is an attack that occurs when a person impersonates a trusted contact or brand, pretending to be someone who is trusted in order to acquire sensitive personal information. This can be used in order to gain access to sensitive personal information.
Fuzzy Attack on the CAN	For the purpose of performing a fuzzy attack on Internet Explorer, for example, a hacker may run Microsoft’s browser inside a debugger tool, allowing them to monitor each command that the application really performs in the memory of the machine.

**Table 3:** Dataset features.

Feature	
Timestamp	time (s) Stamp
Time of the CAN	CAN message (HEX (ex. 043f))
Data length code (DLD) of the CAN	Bytes of data, from 0 to 8
DATA of the CAN [0~7]	Values of data (bytes)

**Table 4:** Results of the KNN model.

**Car-Hacking Dataset**			
Attacks	Precision %	Recall %	F1-score %
#Flooding_attack	100	100	100
#Fuzzing_attack	99	90	94
#Normal	99	100	99
#Spoofing (gear)_attack	81	43	56
#Spoofing Attack (RPM)_attack	100	100	100
Accuracy 98.80			
Weighted average	99	99	99
**UNSW Dataset**			
Attack	Precision %	Recall %	F1-score %
#Analysis_attack	100	100	100
#Backdoor_attack	0.00	0.00	0.00
#DoS_attack	100	100	100
#Exploits_attack	100	100	100
#Fuzzers_attack	48	52	50
#Generic_attack	99	99	99
#Normal_attack	100	100	100
#Reconnaissance_attack	55	54	55
#Worms_attacks	0.00	0.00	0.00
Accuracy 97.37			
Weighted average	97	97	97

**Table 5:** Results of the decision tree model.

**Car-Hacking Dataset**			
Attack	Precision %	Recall %	F1-Score %
#Flooding_attack	100	100	100
#Fuzzing_attack	98	94	95
#Normal	99	100	99
#Spoofing (gear)_attack	72	48	58
#Spoofing Attack (RPM)_attack	100	100	100
Accuracy 99			
Weighted average	99	99	99
**UNSW-NB15 Dataset**			
Attack	Precision %	Recall %	F1-score %
#Analysis_attack	100	100	100
#Backdoor_attack	0.08	0.06	0.07
#DoS_attack	100	100	100
#Exploits_attack	100	100	100
#Fuzzers_attack	50	39	44
#Generic_attack	98	99	99
#Normal_attack	100	100	100
#Reconnaissance_attack	54	56	55
#Worms_attacks	0.05	0.07	0.06
Accuracy 97.19			
#Weighted average	97	97	97

**Table 6:** Results of deep learning.

**Car-Hacking Dataset**				
Model	Accuracy	Precision %	Recall %	F1-score %
LSTM	96.03	96.18	96.17	96.82
Autoencoder	99.98	99.96	99.85	99.96
**UNSW-NB15 Dataset**				
Model	Accuracy	Precision %	Recall %	F1-score %
LSTM	97.82	97.26	98.69	97.97
Autoencoder	98.09	98.12	98.04	98.08

**Table 7:** Statistical analysis.

**Car-Hacking Dataset**			
Model	MSE	RMSE	R^2^ %
KNN	0.01200	0.01210	94.61
Decision tree	0.01178	0.01190	94.70
LSTM	0.0279	0.0136	92
Autoencoder	0.0065	0.0106	95
**UNSW-NB15 Dataset**			
Model	MSE	RMSE	R^2^ %
KNN	0.086	0.1737	86.95
Decision tree	0.1905	0.396	86.17
LSTM	0.0059	0.076	80.87
Autoencoder	0.0052	0.069	92

**Table 8:** Comparison between Car-Hacking and UNSW-NB 15.

References	Car-Hacking Dataset (Accuracy)	UNSW-NB15 Dataset (Accuarcy)
KNN	98.82	97
Decision tree	99	97.19
LSTM	96.03	97.82
Autoencoder	99.98	98.09

**Table 9:** Performance of the proposed system in comparison with that of other research on intrusion detection systems for in-vehicle networks.

References	Algorithms	Model (Accuracy %)	Number of Attacks
Ref. [52]	CNN-LSTM	97%	4 attacks and normal
Ref. [55]	LSTM model	80%	4 attacks and normal
Ref. [56]	ML	90%	4 atatcks and nromal
Ref. [57]	Neural network and LSTM	90%	3 atatcks and normal
Our system	Deep autoencoder	99.98%	4 attacks and normal

## Data Availability

https://ocslab.hksecurity.net/Datasets/car-hacking-dataset (Accessed date 20 December 2022), https://research.unsw.edu.au/projects/unsw-nb15-dataset (Accessed date 29 March 2023).
